# Accepting Muslim minority practices: A case of discriminatory or normative intolerance?

**DOI:** 10.1002/casp.2450

**Published:** 2020-01-16

**Authors:** Sander Sleijpen, Maykel Verkuyten, Levi Adelman

**Affiliations:** ^1^ Ercomer, Utrecht University Utrecht The Netherlands

**Keywords:** discrimination, Muslims, norms, tolerance

## Abstract

West European societies have seen strong debates about the acceptance of Muslim minority practices. In the current research we sought to better understand intolerance by examining whether people use a double standard in which the same practices are tolerated of Christians but not of Muslims (discriminatory intolerance), or rather reject the practices independently of the religious minority group because these are considered to contradict society's normative ways of life (normative intolerance). The results of two survey‐embedded experiments among native Dutch were most in agreement with an interpretation in terms of normative intolerance rather than discriminatory intolerance. This suggests that the rejection of Muslim practices has less to do with Muslims per se but rather with the perceived normative deviance of the practices, independently of the religious minority group. These findings broaden the research on anti‐Muslim sentiments and thereby the debate on the place of Islam within Western liberal societies.

## 1 INTRODUCTION

Historically in Europe, the concept of tolerance evolved as a way to manage the religious conflicts of the sixteenth and seventeenth century (Walzer, 1997). In a modern variation on this, the presence of Muslims in Western Europe has led to strong public debates on the building of Mosques and minarets, the founding of Islamic schools, the wearing of headscarves in public places, and various other Muslim practices^1^ (Helbling, 2012). It is around these concrete issues that religious and cultural diversity is put to a test and ways of life collide.

While some natives in West European societies are tolerant of Muslim practices, others are found to be rather intolerant, and there are two competing explanations for this intolerance. The first explanation focuses on prejudicial attitudes towards Muslims as a group and Islam as a religion. Several studies in Western Europe conclude that intolerance towards particular practices (e.g. wearing of a headscarf) is driven by dislike of Muslims as a group (e.g. Helbling, 2014; Saroglou, Lamkaddem, Van Pachterbeke, & Buxant, 2009). The second explanation focuses instead on the nature of the practice. Being intolerant towards specific practices (e.g. ritual slaughter of animals) does not always coincide with being prejudiced towards Muslims (e.g. Adelman & Verkuyten, 2019; Sniderman & Hagendoorn, 2007).

With the first explanation, we can expect people to apply a double standard, for example by accepting practices of minority groups that are historically established and aligned with the national majority (i.e. specific Christian denominations in Western countries) but not of Muslim minorities engaging in the same practice (*discriminatory* intolerance; Hurwitz & Mondak, 2002). In the latter case, particular practices might be considered to go against societal conventions and norms and are therefore rejected independently of the religious minority group practicing them (*normative* intolerance).

Similarly, while those motivated by prejudice might be expected to oppose all practices of a minority group, those motivated by a societal norm would not necessarily be expected to oppose all practices in the same way. Thus, the opposition to a particular practice of a religious minority group does not have to imply that other, less normatively dissenting practices of that group are also rejected. A person tolerating the wearing of religious symbols might not accept Islamic education, and tolerating Islamic education does not have to mean that one tolerates the building of Mosques (Adelman & Verkuyten, 2019).

The aim of the present study is to compare tolerance of concrete practices performed by either Muslims or Christians to test whether majority members demonstrate discriminatory intolerance or rather normative intolerance. We conducted two survey‐embedded experiments among samples of Dutch natives. In the first experiment, the practices were either performed by immigrant groups of Turkish Muslims or Turkish Christians, while in the second experiment the actors performing the practices were either Muslims or Orthodox‐Protestants in the Netherlands. This allows us to investigate whether people are discriminatory intolerant towards Muslims (compared to Christians) or demonstrates normative intolerance independent of the religious minority group, and whether this depends on the specific religious practices.

## 2 DISCRIMINATORY AND NORMATIVE INTOLERANCE

Following the classical understanding of tolerance in the philosophical and political science literature, we conceptualize tolerance as putting up with something that one is negative about (Gibson, 2006; Horton, 1996). This understanding is also proposed in social psychology (Verkuyten & Yogeeswaran, 2017) and implies acceptance of particular minority practices *despite* having negative attitudes. The negativity aspect in tolerance can be based on disapproval of a practice itself or rather on dislike of the minority group performing it (Hurwitz & Mondak, 2002). Thus the source of intolerance may emerge either from disapproval of a given practice or from group‐based prejudice.

In support of the second explanation, evidence suggests that people might be intolerant of particular practices because they are prejudiced towards Muslims as a group. Several studies in Western Europe conclude that the rejection of a particular practice is associated with and driven by dislike of Muslims. For example, prejudice has been found to underlie the willingness to ban the wearing of the headscarf (Helbling, 2014; Saroglou et al., 2009; Van der Noll, 2014). In support of the first explanation, other studies demonstrate that the relationship between prejudice towards Muslims and acceptance of their religious practices is not that straightforward. Some individuals do not dislike the group of Muslims, but still oppose particular practices, while others dislike the group but accept some practices. For example, by using an unobtrusive measure of prejudices it has been shown in the Netherlands (Sniderman & Hagendoorn, 2007) and in Canada (Breton & Eady, 2015) that the rejection of Muslim practices can be based on the disapproval of the practice itself rather than prejudice towards Muslims as a group of people (see also Adelman & Verkuyten, 2019; Bilodeau, Turgeon, White, & Henderson, 2018). Further, an experimental research in the U.K. demonstrated that people's concerns about Muslim immigrants can be more about strict forms of religiosity that are seen as incompatible with western liberal norms and values then about Muslim immigrants as a group (Helbling & Traunmüller, 2018).

One way to distinguish between discriminatory and normative intolerance is to look for double standards in people's expressions of intolerance. With discriminatory intolerance we can expect people to show a double standard in which the same practice is tolerated in the case of one religious minority group but not in the case of another minority group, whereas with normative intolerance the rejection of the same practice should be independent of the religious minority group (Helbling & Traunmüller, 2018). To utilize this distinction, the current research uses either Christian or Muslim minorities performing the same religious practices. By comparing tolerance of the same practices performed by these two religious minority groups, the relevance of the religious group and the specific practices people are asked to tolerate can be separated. Specifically, in Experiment 1, we compared tolerance of practices of Turkish Muslim immigrants and Turkish Christian immigrants. This comparison is possible because in the 1970s and 1980s not only Muslims but also Christians emigrated from Turkey to the Netherlands. The comparison allows us to examine whether people are less tolerant towards Muslims compared to Christians of the same immigrant‐origin group. A difference in tolerance would indicate the use of a double standard against Muslim compared to Christian Turkish immigrants.

However, the fact that both have emigrated from the same country might be more important than their specific religious background. As a result, people might be equally (in)tolerant towards Turkish immigrants independently of whether they are Muslim or Christian (Spruyt & Elchardus, 2012; Strabac & Listhaug, 2008). Yet Muslims might be tolerated less compared to non‐immigrant religious minority groups. Therefore, in Experiment 2 we examined tolerance towards Muslims and Orthodox Protestants as two numerically small religious groups in the Netherlands (both representing around 5% of the Dutch population) that both struggle to hold on to their religious values and beliefs in the increasingly secular context of the Netherlands (Fetzer & Soper, 2003; Ribberink, Achterberg, & Houtman, 2017). This comparison allows us to assess whether people apply a double standard by tolerating practices of a historically established religious minority group aligned with the national majority (i.e. Orthodox Protestants in the Netherlands) but not of the Muslim minority who might be perceived as threatening the Christian aspect of the national identity (Vollaard, 2013).

Additionally, double standards of tolerance can be applied only regarding a particular practice or rather consistently across multiple practices. Considering different practices is therefore important for assessing whether tolerance of the two religious groups is practice‐specific or generalizes across practices. Not all dissenting practices are equally controversial or normatively dissenting (Adelman & Verkuyten, 2019; Hurwitz & Mondak, 2002), and differential tolerance of various practices without applying a double standard suggests a more principled position of normative tolerance. In both experiments we used the same four practices that have been debated in the Dutch public sphere and that vary in the degree to which they evoke negative feelings and are considered to contradict the Dutch majority's normative way of life (Gieling, Thijs, & Verkuyten, 2010; Hirsch, Verkuyten, & Yogeeswaran, 2019).

## 3 EXPERIMENT 1

### 3.1 Data and method

Data for this research were collected in early February 2018. An online questionnaire was distributed among native Dutch adults via email by ThesisTools, a quantitative research platform specialized in collecting data (similar to MTurk). The questionnaire contained the two survey embedded vignette experiments (1 and 2) used for this research and each experiment was presented to a separate subsample.

The initial subsample of Experiment 1 consisted of 402 participants who were randomly assigned to the two experimental conditions. Since the present study focused on tolerance judgements among native Dutch adults, only participants who were 18 years of age or older and who indicated that they themselves and both of their parents had a Dutch background were included, leading to seven participants being excluded from analyses. Participants who did not answer at least one of the questions about tolerance were also excluded (*N* = 33), as was one participant who did not give their consent to participate. This resulted in a final sample size of 361 participants who were 50% female, ranged in age from 19 and 91 years (*M* = 57.9, *SD* = 14.7), and more than half of whom (56%) were enrolled in or had completed education at the higher vocational or university level. On average, respondents identified themselves as slightly left‐leaning on the well‐known political self‐placement scale (Jost, 2006:22% left; 24% centre‐left, 30% centre, 17% centre‐right, 7% right). In total, 53% of the participants indicated that they were not affiliated with any religion and 46% was affiliated with a Christian denomination (Catholic Church, Protestant Church the Netherlands, Liberal Protestant, Evangelical‐Reformation, or other small Christian groups).

In line with recent research on tolerance of Muslims (e.g. Aarøe, 2012; Gieling et al., 2010; Hirsch et al., 2019), an experimental vignette design was employed to assess tolerance towards concrete and socially relevant situations. Vignettes make it possible to include contextual factors to make the situation more realistic and less abstract (Steiner, Atzmüller, & Su, 2016). This is important when studying tolerance since people are more likely to support the notion of tolerance in general than in relation to concrete situations. Moreover, vignettes reduce socially desirable answers when investigating socially sensitive topics (Auspurg, Hinz, Sauer, & Liebig, 2014).

In order to compare tolerance judgements towards Turkish Muslims and Turkish Christians, as well as Orthodox Protestants in Experiment 2, the dissenting or controversial practices presented in the vignettes needed to be relevant and meaningful for the Dutch context but also similarly relevant across these religious minority groups. In addition, in order to be the subject of tolerance (i.e. acceptance of *disapproved* of practices), the practices should elicit negative feelings and be contested by a substantial proportion of the population. With these considerations in mind and based on previous research, four fictional but realistic scenarios were constructed addressing the following topics: the wearing of a religious necklace by a civil servant, the organization of religious lessons in a community centre, the request of a quiet room at the workplace for praying, and a proclamation by a religious authority person in which he equates abortion with murder. Comparable situations have been used in previous studies on tolerance of Muslims (e.g. Aarøe, 2012; Gieling et al., 2010; Hirsch et al., 2019; Verkuyten & Slooter, 2007).

All four vignettes were presented to each participant in random order, which prevented any sequence effect from the scenarios (Auspurg & Jäckle, 2017). After reading each vignette, respondents were asked the same tolerance question about the scenario they had just read. Each respondent received the same four scenarios, but the actor performing the controversial practice was manipulated and held constant for each participant, following a two (religious affiliation of the target group; between subjects) by four (scenarios; within subjects) mixed experimental design. This resulted in four different versions to which participants were randomly assigned. The vignettes were presented as follows, although not in this fixed order:

1. Recently, there has been a public debate whether the municipality should allow one of their [Turkish Christian/Turkish Muslim] front desk clerks to visibly wear a necklace with a [Christian cross/Islamic crescent moon].
2. Recently there has been a public debate over [Turkish Christians/Turkish Muslims] who would like to organize religious lessons in a community centre. During these lessons, [strict Christian/Islamic] values will be taught.
3. Recently, there has been a public debate over the request of [Turkish Christian/Turkish Muslims], who would like to set up a quiet room at work. All employees would be able to use this room for prayer.
4. Recently, there has been a public debate over an [Turkish Christian minister/Turkish Muslim imam] who would like to have the freedom to give a speech at an educational college in which he equates abortion with murder.

### 3.2 Measures

Tolerance was measured by asking respondents what they thought the authority (depending on the vignette, either the municipality, board of the community centre, employer, or school board) should do in each scenario. Participants could answer these questions with one of the following answer categories that have been successfully used and analysed in previous research (e.g. Gieling et al., 2010; Hirsch et al., 2019; Verkuyten & Slooter, 2007): do nothing and allow it (1), try to convince them not to do it, but allow it if they do not agree (2), try to convince them not to do it, but forbid it if they do not agree (3) and forbid it (4). Items were subsequently reverse coded so that higher values corresponded to more tolerant attitudes.

Participants also indicated their feelings towards each scenario (1 ‘very negative’ to 7 ‘very positive’) which allows us to investigate if the reactions to the scenarios are indeed a matter of tolerance and whether the four scenarios differ in the negative feelings that they elicit.

The degree to which participants considered each practice as contradicting societal norms was measured by using a seven‐point scale ranging from *strongly disagree* (1) to *strongly agree* (7). This single‐item measure was based on the work of Skitka, Bauman, and Sargis (2005) and directly asked to what extent each practice goes against the Dutch normative way of life.

### 3.3 Analyses

Randomisation checks were conducted in order to determine the necessity of including control variables. This showed that it was not necessary to include gender, education, political orientation, Dutch identification (measured with three items on seven‐point scales; Verkuyten, 2011), and age, as there were no significant differences between the conditions. However, religiosity, measured as the importance of religion or spirituality in daily life on a seven‐point scale, did vary between the conditions in Experiment 1, *F*(1, 336) = 7.367, *p* = .007. And since the controversial practices central to this study were of religious nature, it may be argued that people who are more religious are also more likely to accept religious behaviour of others. Therefore, religiosity was included as a control variable.

Furthermore, we also considered religious affiliation. Following Social Identity Theory (Tajfel & Turner, 1986), in‐group bias may affect the acceptance of dissenting practices performed by people with the same religious background. This means that participants who self‐identify as Christians might be more likely to be tolerant towards Turkish Christians than towards Turkish Muslims. Therefore, religious affiliation was included as a dichotomous variable indicating whether participants were (1) or were not (0) affiliated with a Christian denomination. Finally, the questionnaire contained one other, unrelated experiment and the sequence of the two experiments was reversed in half of the questionnaire. In order to take into account potential carry‐over effects (Brooks, 2012) a dichotomous variable was created (1 = *second experiment in questionnaire*, 0 = *first experiment in questionnaire*) and added as a control.

The Statistical Package for the Social Sciences (SPSS) version 21 was employed for data preparation and preliminary analyses. Mplus (Version 7.3, Muthén & Muthén, 2012) was used to test the proposed relationships by conducting a multivariate regression analysis on tolerance for the four scenarios. Since the variables measuring tolerance were relatively skewed, the MLR estimator was applied to correct standard errors for non‐normal distributions. Missing values were minimal for most variables and were dealt with by Mplus by full information maximum likelihood assuming the missing values were missing at random (Muthén & Muthén, 2012).

In order to use multivariate regression analysis, the dependent variables should be measured at the interval or ratio level (Agresti & Finlay, 2009). Although strictly speaking the scale measuring tolerance consisted of four answer categories which are logically arranged in a meaningful order, the scale has been analysed as continuous in previous research (e.g. Gieling et al., 2010; Hirsch et al., 2019; Verkuyten & Slooter, 2007). Therefore, in this study tolerance was also treated as a continuous measurement in the main analysis. In addition, the variances and covariances of the four tolerance variables were constrained to be equal across the groups in order to increase statistical power, since a Box's M test of equality of covariance in SPSS was non‐significant, *F* (10, 520,395.930) = .835, *p* = .595. Lastly, since tolerance towards the four practices was dependent on each other (i.e. all four situations were presented to each participant), correlated uniqueness of repeated measures design was applied, allowing for correlations between the error terms of the variables measuring tolerance (Kline, 2016). The religious affiliation of the target group of the vignettes was included as a dummy variable (1 = *Muslim with a Turkish background*, 0 = *Christian with a Turkish background*).

### 3.4 Results

The question of tolerance occurs when people disapprove of certain practices and, therefore, we first examined to what extent the four practices evoked negative feelings (see Table 1). On average, participants evaluated the four situations negatively as the mean scores were all significantly below the neutral midpoint of the scale (all *p*_s_ < .01). Additionally, there was variation in the degree to which the different situations were evaluated negatively. The anti‐abortion speech evoked the most negative feelings and all mean scores except for the wearing of a religious necklace by a civil servant and request of a prayer room at the workplace, differed significantly from each other (see Table 1).

**Table 1:** Means and *SD*s for negative feelings, contrasting societal norms and tolerance of the four scenarios in Experiment 1 and Experiment 2

	Necklace	Lessons	Prayer room	Anti‐abortion	*F*	*n* ^2^
Experiment 1						
Feeling	3.57 (1.43)_a_	2.94 (1.34)_b_	3.49 (1.80)_a_	2.07 (1.53)_c_	80.19**	.20
Social norm	3.66 (2.03)_a_	4.37 (1.83)_b_	4.68 (1.98)_b_	5.30 (1.70)_c_	57.83**	.16
Tolerance	2.90 (1.24)_a_	2.76 (1.23)_a,b_	2.63 (1.29)_b_	2.18 (1.25)_c_	25.66**	.07
Experiment 2						
Feeling	3.67 (1.38)_a_	3.12 (1.35)_b_	3.56 (1.66)_a_	1.99 (1.44)_c_	132.30**	.27
Social norm	3.44 (1.83)_a_	4.26 (1.70)_b_	4.23 (1.94)_b_	5.15 (1.71)_c_	68.29**	.17
Tolerance	3.06 (1.19)_a_	2.94 (1.14)_a,b_	2.84 (1.24)_b_	2.25 (1.18)_c_	45.56**	.11

*Note*: One‐way correlated analyses of variance. Mean scores with the same subscripts do not significantly differ across the situations at the .05 level after Bonferroni correction. Negative feelings and contrasting societal norms on 7‐point scale, tolerance on 4‐point scale.

** *p* < .001.

Participants also perceived the four situations as contradicting the Dutch normative way of life (see Table 1). They considered the anti‐abortion statement as going most strongly against societal norms, followed by the organizing of religious lessons and the request of a prayer room at the workplace which evoked similar perceptions of norm deviance, while the wearing of a religious necklace was not considered to go against societal norms.

Next, we examined whether tolerance judgments differed across the four minority practices. On average and despite their negative feelings towards the wearing of a religious necklace, the organization of conservative religious lessons and the request of a prayer room, participants were more likely to accept these practices than to forbid them as the mean scores were significantly above the midpoint of the scale (*p*_s_ < .01). However, participants were rather intolerant towards the anti‐abortion statement made by a religious authority (see Table 1). The four practices varied in the extent to which people tolerated them, meaning that people took into account the specific practice they were asked to tolerate. This was further supported by the low to moderate inter‐correlations between the degree of tolerance of the four practices (*r* < .33).

**Figure 1:**
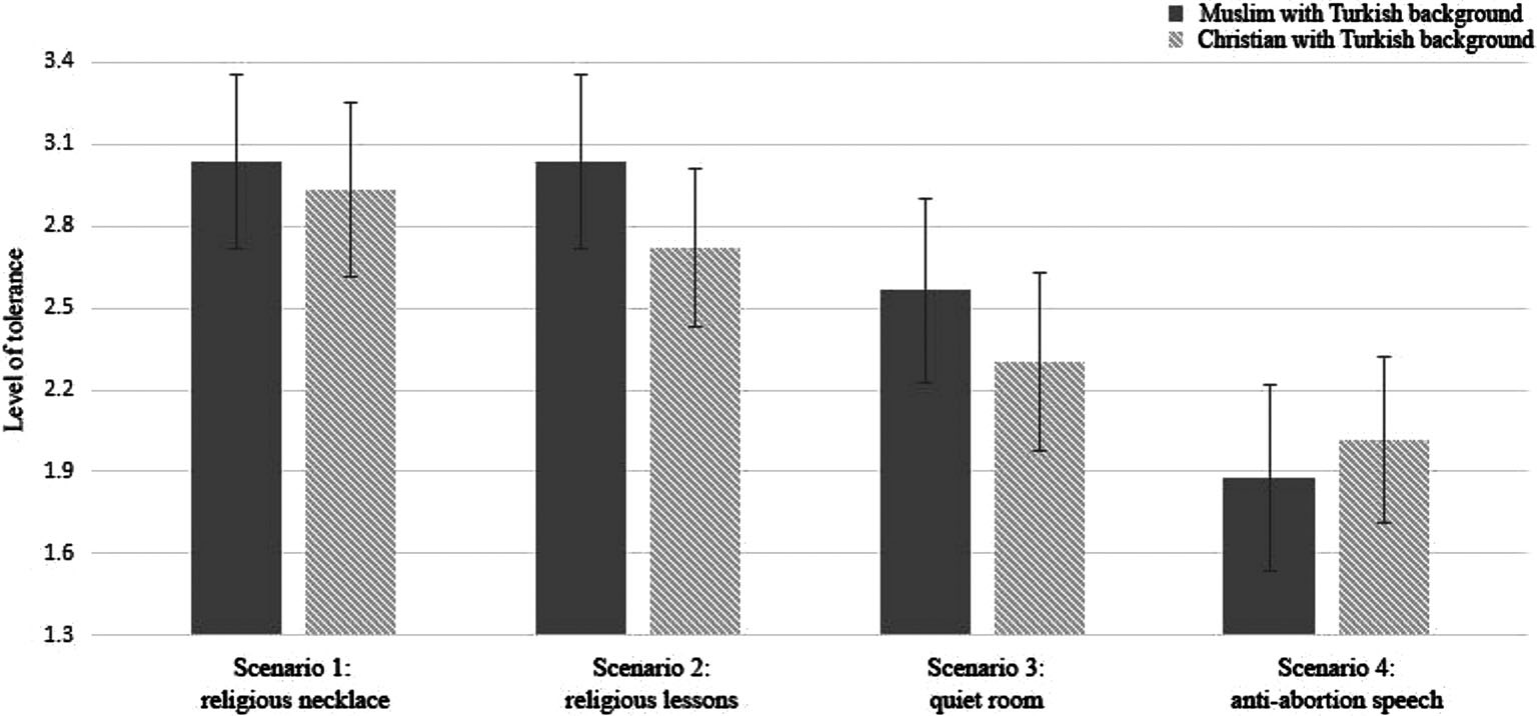
Results from multivariate regression with tolerance for the scenarios as dependent variables controlled for religious affiliation, religiosity and place of the experiment in the questionnaire (Experiment 2). Only mean tolerance scores are depicted. The 95% confidence intervals are represented in the figure by the error bars attached to each column

Results of a multivariate regression analysis^2^ of tolerance towards each scenario on target group revealed that Muslims and Christians with a Turkish background did not evoke significantly different levels of tolerance for any of the four practices (see Figure 1 and Table 2). This indicates that whether or not one tolerates these various practices did not depend on the religious background of the Turkish immigrants.

**Table 2:** Multivariate regression with tolerance for the scenarios as dependent variables (Experiment 1; *N* = 337)

	Scenario 1: Religious necklace		Scenario 2: Religious lessons		Scenario 3: Prayer room		Scenario 4: Anti‐abortion speech	
	*b*	*SE*	*b*	*SE*	*B*	*SE*	*b*	*SE*
Target group								
Muslims with a Turkish background^a^	.116	.184	.317	.176	.262	.192	−.137	.188
Religious affiliation								
Christian^b^	.048	.209	.070	.210	.247	.227	−.298	.201
Christian * target group	−.286	.265	−.358	.268	−.636*	.283	.050	.264
Control variables								
Religiosity	.046	.040	.025	.039	.085*	.041	.171***	.037
Reversed order	−.270*	.134	−.257	.133	.059	.140	−.278*	.131
Intercept	2.932***	.163	2.719***	.149	2.302***	.167	2.016***	.156
Log likelihood (*df*)	−2,122.287 (34)							

a Reference category: Christians with a Turkish background.

b Reference category: non‐Christian.

* *p* < .05.

*** *p* < .001.

Similarly, participants' self‐identification as Christian or having high levels of religiosity did not affect tolerance differently when the practice was performed by a Muslim or a Christian with a Turkish background (see Table 2). Self‐identifying as Christian was not associated with higher levels of tolerance towards the four practices, while those participants for whom religion was more important were more likely to tolerate the request for a prayer room and the anti‐abortion speech, independent of the religious actor. This again supports the notion that controversial practices performed by either Christians or Muslims with a Turkish background were equally accepted.

Analyses of covariance for the four practices separately with target group as a between‐subjects factor and social norm deviation as a covariate showed that stronger belief that a particular practice goes against the Dutch way of life was significantly associated with lower tolerance for all four practices (all *B*s > −.29 and <−.47). Furthermore, this association was similar for Turkish Christians and Turkish Muslims across the practices, except for religious lessons in the community centre, *F*(1, 329) = 4.88, *p* = .028, partial eta squared = .015. For the latter practice, the association was somewhat stronger in relation to Turkish Muslims, *B* = −.43, *t* = 10.33, *p* < .001, 95%CI [.34, .51], than Turkish Christians, *B* = −.29, *t* = 6.11, *p* < .001, 95%CI [.20, .38].

### 3.5 Robustness checks

We conducted two additional analyses to examine the robustness of the findings. First, treating an ordinal variable with less than 5 answer categories as continuous may lead to biased parameter estimates (e.g. Jamieson, 2004; Rhemtulla, Brosseau‐Liard, & Savalei, 2012). Therefore additional analyses were conducted to check whether the results were sensitive to the treatment of tolerance as a continuous measure. A common ordinal measurement scale approach that compared the levels of tolerance and the effects of the covariates across groups and scenarios showed similar results (see Supporting Information).

Second, because tolerance implies a negative attitude and some of the participants did not report negative feelings towards the scenarios, we additionally examined the level of tolerance for only the participants who reported negative feelings. These additional analyses showed that the findings were similar to those of the main analyses (see Supporting Information).

## 4 EXPERIMENT 2

The results of Experiment 1 show that participants had equal levels of tolerance towards the four practices for Turkish Muslims compared to Turkish Christians. This indicates that participants did not use a double standard in their tolerance of Muslim minorities. However, this finding might be due to the fact that both religious groups are Turkish immigrants and that their shared immigrant status is considered more important than their different religious backgrounds. It could be that Muslims are tolerated less compared to non‐immigrant religious minority groups. Therefore we examined in Experiment 2 whether tolerance judgements of the controversial practices differed when they were performed by Dutch Muslims compared to Dutch Orthodox Protestants.

### 4.1 Data and method

The original sample consisted of 439 participants who had not taken part in the first experiment. Again, non‐Dutch participants and those younger than 18 years, were excluded (*N* = 10), along with those who did not answer at least one of the questions about tolerance (*N* = 33), and three participants did not give their consent for the survey. Two other participants were not included in the analyses because they self‐identified as Muslim. This resulted in a final sample size of 381 participants which was 44% female, ranged in age from 19 to 89 years (*M* = 56.7, *SD* = 15.1), and where more than half of the participants (64%) was enrolled in or had completed education at the higher vocational or university level. On average, the sample was slight left‐leaning in political terms (22% left; 19% centre‐left, 37% centre, 15% centre‐right, 7% right). Of the participants 57% indicated no affiliation with a religion and 42% was affiliated with a Christian denomination.

The same experimental design and the same vignettes were used in Experiment 2. The only difference was that the groups performing the dissenting practices were either Muslims or Orthodox Protestants living in the Netherlands. Tolerance judgements, feelings towards the practices, and the degree to which they were perceived to contradict the Dutch normative way of life were also measured in the same ways as in Experiment 1.

### 4.2 Analysis

As in Experiment 1, randomisation checks showed that it was not necessary to include gender, education, and political orientation. Also, the average age, Dutch identification, and the importance of religion did not vary significantly between the conditions. This meant that all experimental conditions were balanced in terms of these demographic participant characteristics, and inclusion of these control variables was therefore not necessary.

As in Experiment 1, religious affiliation and the place of the experiment were added as additional controls. After examining and recoding some of the ‘other’ responses that nevertheless fit in one of the existing categories, a dichotomous variable was created indicating whether someone was affiliated with an Orthodox‐Protestant church. Following the classification of Stoffels (2008), people belonging to the Protestant denominations of Evangelical‐Reformation and the so‐called ‘*Bevindelijk gereformeerd*’ (‘reformed’) were considered Orthodox‐Protestants.

The method of analysis for Experiment 2 was exactly the same as for Experiment 1. Again, the variances and covariances of the four tolerance variables were constrained to be equal across the groups, since a Box's M test of equality of covariance in SPSS was non‐significant, *F*(10, 609, 180.267) = 1.422, *p* = .163. The religious affiliation of the target group of the vignettes was included as a dummy variable (1 = *Muslims*, 0 = *Orthodox Protestants*).

### 4.3 Results

The feelings towards the four practices were similar as in Experiment 1 (see Table 1). On average, participants evaluated the four situations negatively as the mean scores were all significantly (*p*_s_ < .01) below the neutral midpoint of the scale. Additionally, there was again variation in the degree to which the different situations were evaluated negatively, with feelings towards the anti‐abortion speech evoking the most negative feelings followed by religious lessons in the community centre, and the scenarios about the wearing of religious symbols and the prayer room at work which did not differ significantly from each other.

Participants also perceived the four situations as contradicting the Dutch way of life (see Table 1). They again considered the anti‐abortion statement as going most strongly against societal norms, followed by the organizing of religious lessons and the request of a prayer room at the workplace, which evoked similar perceptions of norm deviance. The wearing of a religious necklace was not considered to go against societal norms.

The practices further varied in the extent to which people tolerated them. Overall, participants showed relatively high levels of acceptance (see Table 1). Again, and despite participants' negative feelings they were, on average, more likely to tolerate the practices than to forbid them as the mean scores were significantly above the midpoint of the scale (*p*_s_ < .01). Tolerance of the religious necklace was significantly higher than of the prayer room and participants were rather intolerant towards the anti‐abortion statement made by a religious authority. The pattern of findings indicates that participants took into account the nature of the practice they were asked to tolerate and this was further indicated by the low to moderate inter‐correlations between the tolerance of the four practices (*r* < .32).

**Figure 2:**
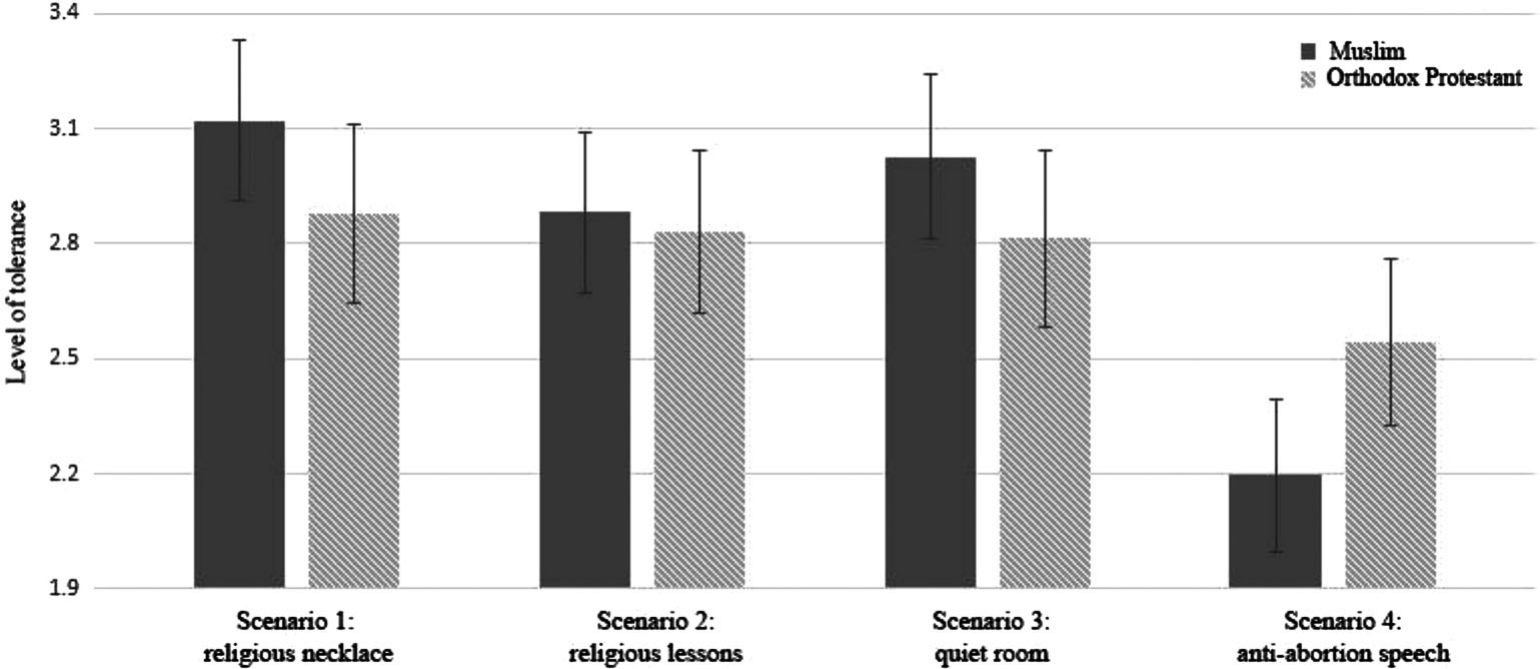
Results from multivariate regression with tolerance for the scenarios as dependent variables controlled for religious affiliation and place of the experiment in the questionnaire (Experiment 1). Only mean tolerance scores are depicted (see Table 3 for more results). The 95% confidence intervals are represented in the figure by the error bars attached to each column

Results of a multivariate regression analysis^3^ of tolerance towards each scenario on target group (i.e. Orthodox‐Protestant or Muslims) showed that target group was only considered relevant for the practice that was evaluated most negatively (see Figure 2 and Table 3). Participants were significantly more tolerant towards an Orthodox‐Protestant minister than an imam giving an anti‐abortion speech, but they were equally tolerant towards an Orthodox‐Protestant or a Muslim in the case of the other three practices.

**Table 3:** Multivariate regression with tolerance for the scenarios as dependent variables (Experiment 2; *N* = 360)

	Scenario 1: Religious necklace		Scenario 2: Religious lessons		Scenario 3: Prayer room		Scenario 4: Anti‐abortion speech	
	*b*	*SE*	*b*	*SE*	*b*	*SE*	*b*	*SE*
Target group								
Muslim^a^	.246	.129	.051	.125	.212	.133	−.348*	.125
Religious affiliation								
Orthodox‐Protestant^b^	.801***	.249	.806**	.257	.143	.472	1.603***	.152
Orthodox P. * target group	−.874*	.431	−.659	.379	.048	.570	−.479	.285
Control variables								
Reversed order	.097	.125	.150	.120	−.110	.129	−.362**	.119
Intercepts	2.876***	.118	2.831***	.108	2.815***	.118	2.543***	.112
Log likelihood (*df*)	−2,209.889 (30)							

a Reference category: Orthodox‐Protestant.

b Reference category: non‐Orthodox‐Protestant.

* *p* < .05.

** *p* < .01.

*** *p* < .001.

Furthermore, participants who identified as Orthodox‐Protestants showed significantly higher levels of tolerance, except for the request of a prayer room at work, compared to participants who belonged to another religious denomination or who were non‐religious (see Table 3). In addition, a significant negative interaction effect indicated that Orthodox‐Protestants were significantly more tolerant towards Orthodox‐Protestants than towards Muslims in the case of wearing a religious necklace. In the case of the other scenarios, there were no significant differences between the target groups.^4^

Analyses of covariance for the four practices separately with target group as a between‐subjects factor and social norm deviation as a covariate showed that stronger belief that a particular practice goes against the Dutch normative way of life was significantly associated with lower tolerance for all four practices (*Bs*, >−.27, <−.43). Furthermore, this association was similar for Orthodox Protestants and Muslims, except, again, for religious lessons in the community centre, *F*(1, 357) = 4.28, *p* = .039, partial eta squared = .012. The latter association was somewhat stronger in relation to Muslims, *B* = −.34, *t* = 8.54, *p* < .001, 95%CI [.26, .48], than for Orthodox Protestants, *B* = −.22, *t* = 4.74, *p* < .001, 95%CI [.13, .30].

Similar to Experiment 1 we examined the robustness of the findings by, first, checking whether the results were sensitive to the treatment of tolerance as a continuous measure and, second, by focusing only on the participants who reported negative feelings. These additional analyses showed that the findings were similar to those of the main analyses (see Supporting Information).

## 5 DISCUSSION

In two survey‐embedded experiments among native Dutch, we found that, in general, participants did not display different levels of tolerance towards Christians and Muslims (see also Helbling & Traunmüller, 2018). Only in Experiment 2, participants showed somewhat lower levels of tolerance towards Muslims than towards Orthodox Protestants, but only in the case of the anti‐abortion speech. No meaningful differences emerged for the other three scenarios or in Experiment 1. This pattern of findings is most in agreement with an interpretation in terms of normative tolerance rather than discriminatory tolerance. Two other findings support this interpretation. One is that tolerance was lowest for those practices (ant‐abortion speech, prayer room at work) that were considered to go most strongly against the Dutch normative way of life. The other is that for each of the four practices, higher perception of societal norm deviance was associated with lower tolerance. This was found equally for both Muslims and Christians, except for religious teaching at the community centre for which the association was somewhat weaker in relation to Christians than Muslims.

This latter finding suggests that discriminatory tolerance was not completely absent. This is also indicated by the finding in Experiment 2 that people were more tolerant of an anti‐abortion speech of an Orthodox Protestant minister than an imam. It might be that the religious background of the actor is more important when a practice is seen as highly negative and that people are more likely to display anti‐Muslim prejudice when they think that there is a legitimate reason to do so (Crandall, Eshleman, & O'Brien, 2002). Since participants had, on average, a strong negative attitude towards the anti‐abortion speech, this might explain their lower level of tolerance towards Muslims compared to Orthodox‐Protestants. Additionally, and further indicating discriminatory tolerance, is that Orthodox Protestant participants were more tolerant towards Orthodox Protestants wearing a religious necklace than Muslims doing the same. However, for the other three practices these participants did not make a religious target group differentiation.

There are some limitations to the current research that provide directions for future studies. First, based on previous research (e.g. Hirsch et al., 2019; Skitka et al., 2005) we have used single item scales for measuring the different construct. The use of rather simple and straightforward questions reduces the problem of meaning and interpretation inherent in more complex measures and has been shown to have adequate validity and reliability in measuring psychological constructs such as group identification (Postmes, Haslam, & Jans, 2013), personal self‐esteem (Robins, Hendin, & Trzesniewski, 2001), and generalized trust (Lundmark, Gilljan, & Dahlberg, 2016). However, future studies might consider using more extensive measures. Additionally, based on randomisation checks we statistically controlled for (demographic) variables that differed between the experimental conditions and future research might consider additional variables such as intergroup contact experiences and group‐based attitudes. Furthermore, the findings might be specific for the four practices used and therefore not generalizable to other practices. Tolerance is not a global construct but depends on the specific practice that people are asked to tolerate which means that future studies should examine the level of tolerance of other Muslim practices (Adelman & Verkuyten, 2019).

Second, the results might be specific for the Dutch context and to relatively higher educated people. It has been shown that anti‐Muslim attitudes are higher in religious countries than in secularized countries (Ribberink et al., 2017). In addition, in a relatively secular country, such as the Netherlands, religion in general is often viewed unfavorably (Cesari, 2010; Ter Borg, 2008). This might explain why native Dutch have similar levels of intolerance towards Christians and Muslim minority groups. However, a similar result was found in an experimental study in the U.K. (Helbling & Traunmüller, 2018). Yet, in more religious countries, a non‐religious member of society might be more tolerant towards followers of the majority religion than towards other religious minority groups. For example, in Denmark, a country of which more than 80% of the citizens belong to the national church, people were found to be more tolerant towards a judge wearing a Christian compared to an Islamic necklace (Aarøe, 2012). Moreover, in the present study levels of tolerance were relatively high and this might be due to the high percentage of higher educated people who tend to be more tolerant (Vogt, 1997). Hence, future research should investigate to what extent the results generalize to other measures, other practices, other countries and other samples.

In conclusion, the present study has tried to go beyond the relatively extensive research on prejudicial attitudes towards Muslims and Islam in Western societies (e.g. Ribberink et al., 2017; Strabac & Listhaug, 2008) by focusing on specific religious practices that are widely debated in society and by comparing reactions towards Muslims with those towards Christian minority groups. A focus on various practices and a religious minority group comparisons allows us to examine whether the acceptance or rejection of Muslim practices is a form of discriminatory (in)tolerance or rather normative (in)tolerance. Our findings are most consistent with the latter interpretation and therefore suggest that the rejection of Muslim practices has less to do with Muslims per se but rather with the perceived normative deviance of the practices, independently of the religious minority group (Helbling & Traunmüller, 2018; Sniderman & Hagendoorn, 2007). These findings broaden the research on anti‐Muslim sentiments and thereby the larger debate on the place of Islam within Western liberal societies.

## Supporting information


**Data S1** Appendices A–D.

## Data Availability

The original data for this study are safely stored at the special storage facility of the Faculty of Social and Behavioural Sciences, Utrecht University, The Netherlands. The data are available for purposes of replication.

## Endnotes

1 Here we use the term ‘Muslim practice’ to refer to the way the public tends to perceive these practices and without suggesting that these are part of Islam.

2 Christian affiliation and its interaction with the target group, religiosity and place of the experiment in the questionnaire were added to the analysis as controls. The interaction terms between the place of the experiments and target group as well as religiosity and target group for each scenario were non‐significant and, therefore, removed from the final analyses.

3 Orthodox‐Protestant affiliation and its interaction with the target group as well as the place of the experiment in the questionnaire were added to the analysis as controls. The interaction terms between the place of the experiments and target group for each scenario were non‐significant and, therefore, removed from the final analyses.

4 In the case of religious lessons, the sum of the main effect and interaction effect was not significantly different from zero. This means that although Orthodox Protestants were more tolerant towards religious lessons when performed by an Orthodox Protestant compared to non‐Orthodox Protestants, this is not the case when the practice is performed by a Muslim. This is reflected in the non‐significant interaction between Orthodox‐Protestant affiliation and target group.
