# Supplementary material for: Accepting Muslim minority practices: A case of discriminatory or normative intolerance?
Source: J Community Appl Soc Psychol. 2020 Jan 16;30(4):405–18. doi: 10.1002/casp.2450 (PMC7383984; doi:10.1002/casp.2450)
Supplement: Supplementary file 1 — Data S1 Appendices A–D. [file CASP-30-405-s001.docx]

**SUPPLEMENTARY MATERIAL**

**Appendix A**

*Multivariate regression with tolerance for the scenarios as dependent variables, only participants with a neutral or negative stance to each scenario are included (Experiment 1; N=335)*

|  | Scenario 1: religious necklace | | Scenario 2: religious lessons | | Scenario 3: prayer room | | Scenario 4: anti-abortion speech | |
| --- | --- | --- | --- | --- | --- | --- | --- | --- |
|  | *b* | *SE* | *B* | *SE* | *b* | *SE* | *b* | *SE* |
| Target group: |  |  |  |  |  |  |  |  |
| Muslims with a Turkish background^a^ | .109 | .205 | .175 | .191 | .214 | .216 | -.112 | .189 |
| Religious affiliation: |  |  |  |  |  |  |  |  |
| Christian^b^ | .150 | .239 | -.011 | .217 | .136 | .274 | -.352 | .202 |
| Christian*Target group | -.273 | .304 | -.220 | .287 | -.533 | .332 | -.033 | .270 |
| Control Variables: |  |  |  |  |  |  |  |  |
| Religiosity | -.010 | .046 | .005 | .041 | .032 | .051 | .129* | .040 |
| Reversed order | -.250 | .148 | -.288* | .141 | .138 | .157 | -.218 | .140 |
| Intercept | 2.815*** | .178 | 2.766*** | .153 | 1.997*** | .177 | 2.064*** | .166 |
| Log likelihood (*df*) | -1745.543 (35) | | | | | | | |

^a^ Reference category: Christians with a Turkish background. ^b^ Reference category: non-Christian.

* *p* < .05. *** *p* < .001.

**Appendix B**

*Multivariate logistic regression with tolerance for the scenarios as dependent variables (Experiment 1; N=337)*

|  | Scenario 1: religious necklace | | Scenario 2: religious lessons | | Scenario 3: prayer room | | Scenario 4: anti-abortion speech | |
| --- | --- | --- | --- | --- | --- | --- | --- | --- |
|  | *b* | *SE* | *B* | *SE* | *b* | *SE* | *b* | *SE* |
| Target group: |  |  |  |  |  |  |  |  |
| Muslims with a Turkish background^a^ | .763 | .476 | .401 | .329 | .634 | .425 | .219 | .317 |
| Religious affiliation: |  |  |  |  |  |  |  |  |
| Christian^b^ | -.201 | .396 | .083 | 211 | .165 | .308 | -.446 | .270 |
| Christian*Target group | -.054 | .459 | -.577 | .367 | -.632 | .454 | .309 | .390 |
| Control Variables: |  |  |  |  |  |  |  |  |
| Religiosity | .087 | .058 | .034 | .044 | .127* | .063 | .209*** | .057 |
| Reversed order | -.415* | .204 | -.315* | .154 | .037 | .207 | -.359* | .173 |
| χ^2^ (*df*) | 17.513 (45) | | | | | | | |

*Note.* A robust weighted least squares estimator using a diagonal weight matrix (WLSMV) is used.

^a^ Reference category: Christians with a Turkish background. ^b^ Reference category: non-Christian.

* *p* < .05. *** *p* < .001.

**Appendix C**

*Multivariate regression with tolerance for the scenarios as dependent variables, only participants with a neutral or negative stance to each scenario are included (Experiment 2; N=357)*

|  | Scenario 1: religious necklace | | Scenario 2: religious lessons | | Scenario 3: prayer room | | Scenario 4: anti-abortion speech | |
| --- | --- | --- | --- | --- | --- | --- | --- | --- |
|  | *b* | *SE* | *b* | *SE* | *b* | *SE* | *B* | *SE* |
| Target group: |  |  |  |  |  |  |  |  |
| Muslim^a^ | .308* | .142 | -.040 | .133 | .071 | .153 | -.382** | .126 |
| Religious affiliation: |  |  |  |  |  |  |  |  |
| Orthodox-Protestant^b^ | -.881*** | .144 | .011 | .701 | -1.197*** | .278 | 1.184*** | .262 |
| Orthodox P.*Target group | .837* | .417 | .227 | .764 | 1.378* | .597 | -.951 | .574 |
| Control Variables: |  |  |  |  |  |  |  |  |
| Reversed order | .045 | .139 | .186 | .129 | -.113 | .150 | -.356** | .126 |
| Intercepts | 2.744*** | .128 | 2.814*** | .115 | 2.622*** | .135 | 2.533*** | .114 |
| Log likelihood (*df*) | -1886.802 (31) | | | | | | | |

^a^ Reference category: Orthodox-Protestant. ^b^ Reference category: non-Orthodox-Protestant.

* *p* < .05. ** *p* < .01. *** *p* < .001.

**Appendix D**

*Multivariate logistic regression with tolerance for the scenarios as dependent variables (Experiment 2; N=360)*

|  | Scenario 1: religious necklace | | Scenario 2: religious lessons | | Scenario 3: prayer room | | Scenario 4: anti-abortion speech | |
| --- | --- | --- | --- | --- | --- | --- | --- | --- |
|  | *b* | *SE* | *b* | *SE* | *b* | *SE* | *b* | *SE* |
| Target group: |  |  |  |  |  |  |  |  |
| Muslim^a^ | .468 | .296 | -.058 | .187 | .369 | .237 | -.502** | .174 |
| Religious affiliation: |  |  |  |  |  |  |  |  |
| Orthodox-Protestant^b^ | 1.559 | .833 | 1.250* | .527 | .172 | .496 | 2.059** | .695 |
| Orthodox P.*Target group | -1.818 | 1.026 | -1.198 | .724 | .118 | .777 | -.988 | .833 |
| Control Variables: |  |  |  |  |  |  |  |  |
| Reversed order | .139 | .180 | .128 | .128 | -.134 | .168 | -.351** | 121 |
| χ2 (*df*) | 12.028 (41) | | | | | | | |

*Note.* A robust weighted least squares estimator using a diagonal weight matrix (WLSMV) is used.

^a^ Reference category: Orthodox-Protestant. ^b^ Reference category: non-Orthodox-Protestant.

* *p* < .05. ** *p* < .01.
